# Photo‐Disassembly of Membrane Microdomains Revives Conventional Antibiotics against MRSA

**DOI:** 10.1002/advs.201903117

**Published:** 2020-01-27

**Authors:** Jie Hui, Pu‐Ting Dong, Lijia Liang, Taraknath Mandal, Junjie Li, Erlinda R. Ulloa, Yuewei Zhan, Sebastian Jusuf, Cheng Zong, Mohamed N. Seleem, George Y. Liu, Qiang Cui, Ji‐Xin Cheng

**Affiliations:** ^1^ Department of Electrical and Computer Engineering Boston University Boston MA 02215 USA; ^2^ Boston University Photonics Center Boston MA 02215 USA; ^3^ Department of Chemistry Boston University Boston MA 02215 USA; ^4^ State Key Laboratory of Supramolecular Structure and Materials Institute of Theoretical Chemistry Jilin University Changchun 130012 China; ^5^ Collaborative to Halt Antibiotic‐Resistant Microbes (CHARM) Department of Pediatrics University of California San Diego School of Medicine La Jolla CA 92093 USA; ^6^ Division of Infectious Disease Department of Pediatrics Children's Hospital of Philadelphia Philadelphia PA 19104 USA; ^7^ Department Biomedical Engineering Boston University Boston MA 02215 USA; ^8^ College of Veterinary Medicine Purdue University West Lafayette IN 47907 USA; ^9^ Division of Infectious Diseases Rady Children's Hospital San Diego CA 92123 USA

**Keywords:** antibiotic resistance, membrane microdomains, pulsed lasers, *Staphylococcus aureus*, staphyloxanthin

## Abstract

Confronted with the rapid evolution and dissemination of antibiotic resistance, there is an urgent need to develop alternative treatment strategies for drug‐resistant pathogens. Here, an unconventional approach is presented to restore the susceptibility of methicillin‐resistant *S. aureus* (MRSA) to a broad spectrum of conventional antibiotics via photo‐disassembly of functional membrane microdomains. The photo‐disassembly of microdomains is based on effective photolysis of staphyloxanthin, the golden carotenoid pigment that gives its name. Upon pulsed laser treatment, cell membranes are found severely disorganized and malfunctioned to defense antibiotics, as unveiled by membrane permeabilization, membrane fluidification, and detachment of membrane protein, PBP2a. Consequently, the photolysis approach increases susceptibility and inhibits development of resistance to a broad spectrum of antibiotics including penicillins, quinolones, tetracyclines, aminoglycosides, lipopeptides, and oxazolidinones. The synergistic therapy, without phototoxicity to the host, is effective in combating MRSA both in vitro and in vivo in a mice skin infection model. Collectively, this endogenous chromophore‐targeted phototherapy concept paves a novel platform to revive conventional antibiotics to combat drug‐resistant *S. aureus* infections as well as to screen new lead compounds.

## Graphical abstract

Decomposition of staphyloxanthin, a membrane‐bound yellow pigment in *Staphylococcus aureus*, by nanosecond pulsed blue laser induces catastrophic disassembly of its functional membrane microdomains. The photo‐disassembly renders membranes permeable and fluidic with important proteins including PBP2a and efflux pump malfunctioned. As a result, photodisassembly of membrane microdomains revives a broad spectrum of conventional antibiotics against *Staphylococcus aureus*.

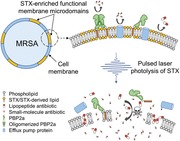

## 1 Introduction

Antibiotic resistance in human pathogens is one of the biggest public health challenges of our time. One such deadly pathogen is *S. aureus* or particularly methicillin‐resistant *S. aureus* (MRSA), which causes high morbidity and mortality worldwide. An estimate of 23 000 fatalities occur each year in the United States due to antibiotic‐resistant infections; surprisingly, nearly half of these deaths (11 285) is due to one bacterial pathogen, MRSA.^1^ The prevalence of its antibiotic resistance is consistently challenging our current treatment options via various molecular mechanisms. Particularly, overexpression of *mecA* encoded penicillin‐binding protein 2a (PBP2a) in MRSA strains reduces the affinity of most beta‐lactams;^2^ active efflux pumps on cell membranes keep intracellular antibiotic concentration at sublethal level, conferring multi‐drug resistance to fluoroquinolones and tetracyclines;^3^ remodeling of membrane composition, for example, phospholipids, reduces the binding thus the effectiveness of daptomycin, a last‐resort antibiotic.^4^ Moreover, the development of new antibiotics is currently unable to keep pace with the emergence of resistant bacteria, thus likely leading us to a post‐antibiotic era.^5^ To tackle this grand challenge, alternative treatment strategies are urgently required.

Grounded on the increasing understanding of virulence factors in disease progression and host defense, anti‐virulence strategies have arisen in the past decade as an alternative.^6^ In *S. aureus*, staphyloxanthin (STX), the yellow carotenoid pigment that gives its name, is one of its important virulence factors.^7^ This pigment is expressed specifically in *S. aureus* for bacterial pathogenesis and used as an antioxidant to neutralize reactive oxygen species (ROS) produced by the host immune system.^8^ Recent studies on cell membrane organization further suggest that STX and its derivatives condense as the constituent lipids of functional membrane microdomains (FMM), endowing membrane integrity and providing a platform to facilitate protein–protein oligomerization and interaction, including PBP2a, to further promote cell virulence and antibiotic resistance.^9^ Therefore, blocking STX biosynthesis pathways has become an innovative therapeutic approach. Thus far, cholesterol‐lowering drugs, including compound BPH‐652 and statins, have shown capability of inhibiting *S. aureus* virulence by targeting the enzymatic activity, for example, dehydrosqualene synthase (CrtM), along the pathway for STX biosynthesis.^9^, ^10^ However, these drugs suffer from off‐target issues, as human and *S. aureus* share the same pathway for biosynthesis of presqualene diphosphate, an intermediate used to produce downstream cholesterol or STX. Additionally, anti‐fungal drug, naftifine, was recently repurposed to block STX expression and sensitize *S. aureus* to immune clearance.^11^ Despite these advances, all of these are still drug‐based approaches to inhibit STX virulence, which require additional treatment time, accompanied by serious side effects, show weak activities, and have higher risk for resistance development by targeting a single upstream biosynthetic enzyme, which will eventually prevent their clinical utilization.

Here, we demonstrate STX photolysis‐mediated photodisassembly of membrane microdomains as a novel strategy to sensitize MRSA to conventional antibiotics. This work presents three significant advances over our previous discovery that STX is prone to bleaching by blue light and that STX photolysis sensitizes MRSA to ROS.^12^ First, grounded on the second‐order STX photolysis kinetics, we show that a nanosecond‐pulsed blue laser is able to strip off this pigment with much higher efficiency than with a low‐level light source (e.g., light‐emitting diode (LED)). Second, we show that STX photolysis by pulsed laser dramatically disorganizes and further malfunctions the FMM, as evidenced by increased membrane fluidity, ample membrane permeability, and PBP2a protein detachment. Third, we show that such FMM disruption facilitates intracellular delivery of small antibiotics, membrane insertion of lipopeptides, and attack by penicillins. As a result, photo‐disassembly of FMM restores the susceptibility and inhibits resistance development to a broad category of conventional antibiotics against MRSA. This work further deciphers the structural and functional properties of STX‐enriched membrane microdomains for antibiotic resistance, thus providing a strategy to tackle antibiotic resistance by targeting STX virulence.

## 2 Results

### 2.1 Pulsed Blue Laser Photolysis of Staphyloxanthin

**Figure 1:**
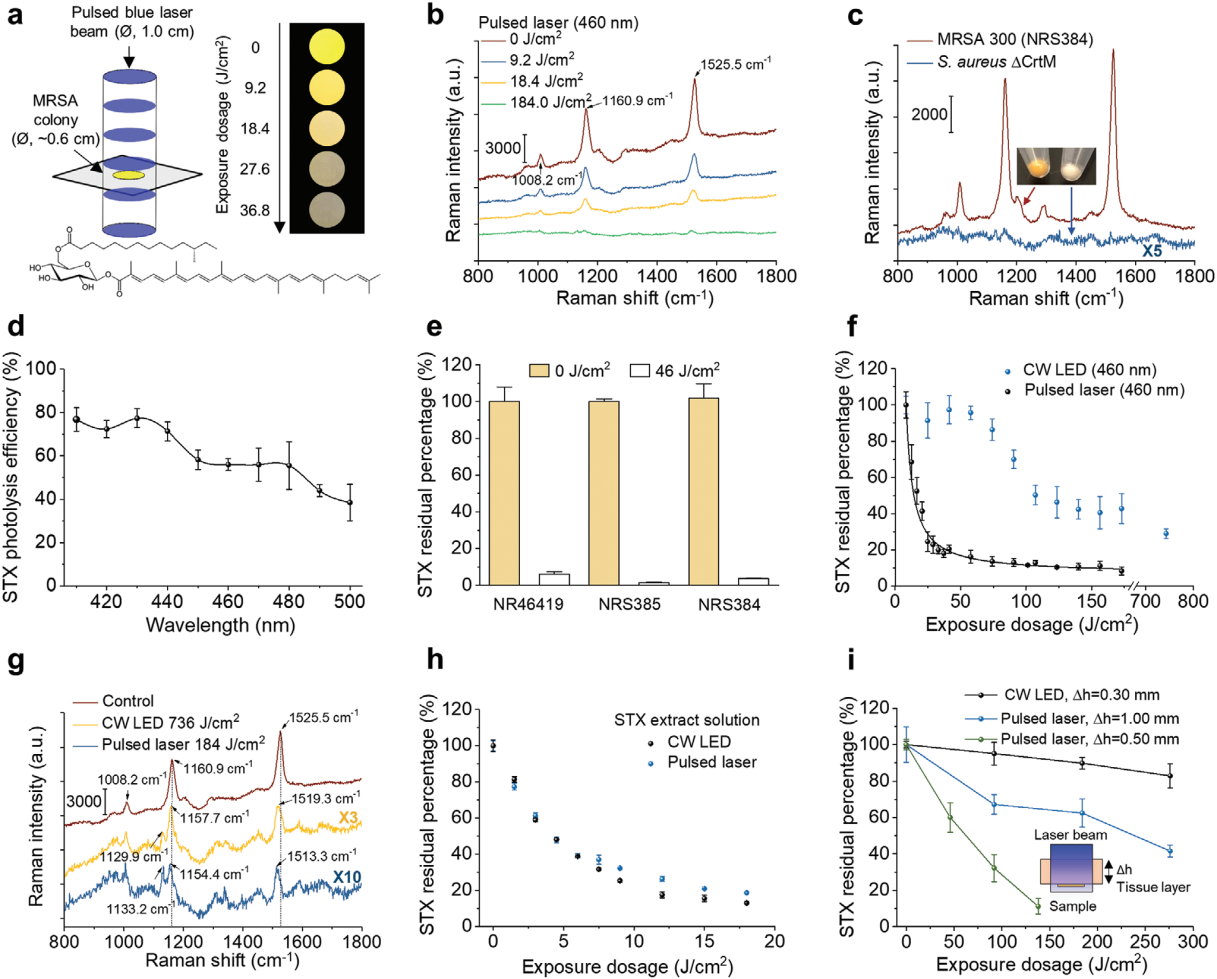
Photophysics and photochemistry of pulsed laser photolysis of STX. a) (Left) Schematic of MRSA (NRS384) colony (or MRSA solution or STX extract solution) treated by nanosecond pulsed laser in a wide‐field illumination configuration. (Right) Digital images of MRSA colony over laser treatment dose or time to show golden color fading phenomenon. Image were recorded with sample placed on a transparent glass cover slide over a black paper. (Bottom) STX molecular structure. Ø refers diameter of bacterial colony. b) Resonance Raman spectroscopy of MRSA (NRS384) colony over 460 nm nanosecond pulsed laser treatment dose (measured on the same colony). Numbers indicate major Raman peak positions. c) Resonance Raman spectroscopy of MRSA (NRS384) and *S. aureus* ΔCrtM colonies. The images show the color of spun‐down cells. d) Spectroscopic study of STX photolysis efficiency in MRSA (NRS384) with nanosecond pulsed laser of the same dose (19 J cm^−2^ with power of 50 mW and treatment time of 5 min). STX photolysis efficiency is quantified by Raman peak amplitude at 1161 cm^−1^. e) Raman quantification of STX abundance in multidrug‐resistant *S. aureus* cells before and after 46 J cm^−2^ laser treatment (460 nm). Bacterial strains include VRSA (NR46419), sulfamethoxazole/trimethoprim‐resistant MRSA (NRS385), and erythromycin‐resistant MRSA (NRS384). f) STX photolysis kinetics of MRSA (NRS384) colony by nanosecond pulsed laser and CW LED under the same illumination power (460 nm). Solid black curve is the fitting result by a second‐order photobleaching model. g) Resonance Raman spectroscopy of STX in MRSA (NRS384) colony with or without high dose‐treatment by nanosecond pulsed laser and CW LED at 460 nm highlighting STX photolysis induced Raman peak shifts and the generation of new Raman peak. Numbers indicate Raman peak positions before and after light treatment. h) STX photolysis kinetics of STX solution by nanosecond pulsed laser and CW LED under the same illumination power (460 nm). STX solution were extracted directly from MRSA cells (NRS384). i) STX photolysis kinetics of MRSA (NRS384) colony placed beneath a tissue layer with various thickness by nanosecond pulsed laser and CW LED under the same illumination power (460 nm). The inset shows the schematic of experimental scheme. Δ*h* indicates the thickness of tissue layer. CW, continuous wave. The cells used were all cultured to reach three‐day stationary phase. *N* = 3 for all the above measurements.

In order to test the hypothesis that STX is the molecular target of photons in the entire blue range, we directly exposed high‐concentration stationary‐phase MRSA (NRS384) colony to a wavelength‐tunable laser beam in a wide‐field illumination configuration as shown in **Figure** **1**a. Strikingly, the distinctive golden color of MRSA colony fades quickly over treatment dose (or time) when the wavelengths were tuned into the blue (400–490 nm) wavelength range (e.g., the images of MRSA colony with 460 nm illumination wavelength in Figure 1a). As the golden colony color is originated from STX pigment, such color‐fading phenomenon suggests that STX is subject to photolysis (molecular structure of STX shown in Figure 1a). In order to further validate this point, we applied resonance Raman spectroscopy to quantify STX content in MRSA cells by taking advantage of its high sensitivity, molecular specificity, and linear concentration dependence.^13^ STX in MRSA (NRS384) shows three characteristic Raman peaks around 1008 (methyl rocking), 1161 (C—C stretch), and 1525 cm^−1^ (C=C stretch), respectively, corresponding to their specific molecular vibrational modes (Figure 1b). With increased laser treatment dose, we observed dramatically decreased peak amplitude for all three Raman bands, suggesting the cleavage of both C—C and C=C bonds that constitutes the polyene chain of STX (Figure 1b). As a result, the unsaturated tail of STX, the nine conjugated C=C double bonds, is decomposed or truncated, as confirmed by mass spectrometry in our previous work.^12^ In contrast, when we blocked STX biosynthesis in wild‐type *S. aureus* by knocking down CrtM, namely *S. aureus* ΔCrtM,^8^ its colony turned colorless and showed no detectable peaks for all three Raman bands, confirming that these Raman bands are exclusively from STX (Figure 1c). With fixed laser power and dose (50 mW, 19 J cm^−2^), MRSA (NRS384) colonies were further illuminated at different laser wavelengths and STX photolysis efficiency calculated using the Raman peak amplitude at 1161 cm^−1^ before and after illumination. The results in Figure 1d indicate that STX is subject to effective photolysis in the entire blue wavelength range (400–490 nm) with significantly reduced efficiency when above 500 nm. This efficiency curve matches the absorption spectrum of STX as photolysis is grounded on the absorption of chromophores (Figure S1a, Supporting Information). The effective STX photolysis induces significant absorption change, which is directly reflected on the absorption spectra of MRSA bacterial solution (Figure S1a, Supporting Information). By compromising the STX photolysis efficiency and optical penetration, 460–480 nm is the preferable optical window (460 nm illumination wavelength was applied in the following studies). Notably, STX photolysis behavior is not only limited to MRSA, but broadly shown on vancomycin‐resistant *S. aureus* (VRSA, NR46419) and other clinically isolated multi‐drug resistant *S. aureus* strains (Figure S1b, Supporting Information, and Figure 1e; their minimum inhibitory concentrations (MIC) shown in Table S1, Supporting Information), as more than 90% of all *S. aureus* human clinical isolates generate this golden pigment.^14^ Collectively, these results suggest that STX is the molecular target of photons or lasers in the entire blue range.

Considering the significance of STX virulence in a MRSA‐caused disease, an optimal light source that enables efficient, fast, complete, and deep depletion of STX is of great importance. Our previous study via transient absorption microscopy suggests that STX photolysis under tightly focused laser primarily follows a second‐order photolysis model due to triplet‐triplet annihilation: T* + T* → R + S, where R and S represent reduced and semi‐oxidized forms.^12^ The triplet excitons form with high yield via singlet fission when carotenoids self‐assemble into multimer or aggregates on cell membrane.^15^ As the triplet lifetime of STX is on a microsecond scale^16^ and STX laterally assembles within FMM,^9^ a nanosecond pulsed laser with high peak power can be used to effectively populate STX molecules to their triplet state within single pulse excitation thus accelerating STX photolysis nonlinearly.

To test this hypothesis, we first exposed stationary‐phase MRSA (NRS384) colony to the nanosecond pulsed laser and a continuous‐wave LED, with output power of 120 mW and wavelength centered at 460 nm, for both light sources, then monitored their residual STX through resonance Raman spectroscopy over different exposure dose. Remarkably, the nanosecond pulsed laser shows unmatched efficiency, speed, and completeness for STX photolysis when compared with the LED, as it depletes 80% of STX in MRSA cells within less than 18 J cm^−2^ exposure dose (2 min exposure time), whereas it takes LED more than 180 J cm^−2^ (20 min) to reach the same efficiency (Figure 1b,f and Figure S1c, Supporting Information); the STX photolysis by LED is not complete even over laser dose of 700 J cm^−2^ (80 min). The efficiency and speed come from the nonlinearity of STX annihilation enabled by nanosecond pulsed laser, consistent with the second‐order fitting^12^ result of the decay curve (Figure 1f). By closely examining the Raman spectra, nanosecond pulsed laser further induces significant blue shifts of these peaks; the shifts are as large as 12 and 6 cm^−1^ for peaks at 1525 and 1161 cm^−1^, respectively (Figure 1g). These blue shifts provide additional evidence to support the photochemistry process in STX. In contrast, when we monitored the photolysis kinetics on STX solution extracted from MRSA pellets, nanosecond pulsed laser and LED no longer show distinctive decay curves (Figure 1h and Figure S1d,e, Supporting Information). Therefore, STX photolysis speed as suggested has high concentration dependence; highly aggregated STX nonlinearly increases STX photolysis efficiency and speed. When laser pulse power was doubled meanwhile keeping exposure dose the same, photolysis delay curves for nanosecond pulsed laser only show minor difference, as likely this two‐time difference in peak power is minor when compared with 10^7^‐time difference between nanosecond pulsed laser and continuous‐wave LED under the same power (Figure S1f, Supporting Information). Thus, further shortened illumination time can be achieved by simply increasing pulse power until reaching saturation. More significantly, the nanosecond pulsed laser enables approximately four‐fold larger treatment depth when compared with LED, as more than 50% STX molecules are depleted by nanosecond pulsed laser when MRSA colonies are placed beneath a tissue layer with thickness beyond 1 mm within one cell cycle (30 min), whereas LED barely penetrates through 300 µm tissue to reach the same efficiency (Figure 1i, experimental schematic shown in the inset). Such effective STX photolysis in deep tissue comes from the conjugation of the photolysis nonlinearity and high peak power of pulsed laser, as the peak power of pulsed laser is several orders of magnitude higher than that of low‐level light sources (e.g., LED). The extended depth is sufficient to penetrate and treat MRSA biofilms (thickness typically ranging from a few micrometers to several hundreds of micrometers^17^), which are normally difficult to treat by antibiotics due to biofilm‐mediated inactivation.^18^ Notably, the illumination power and dose for nanosecond pulsed blue laser in this study are below the American National Standards Institute safety limit for human skin exposure to lasers at 460 nm.^19^ In contrast to continuous‐wave LED, nanosecond pulsed laser further eliminates potential photothermal issues as the temperature rise on human skin is quite small (<5°). Collectively, these results suggest that nanosecond‐pulsed blue laser is the superior light source to deplete STX in MRSA quickly, effectively, completely, and safely.

### 2.2 Photo‐Disassembly of FMM Mechanism 1: Membrane Permeabilization

**Figure 2:**
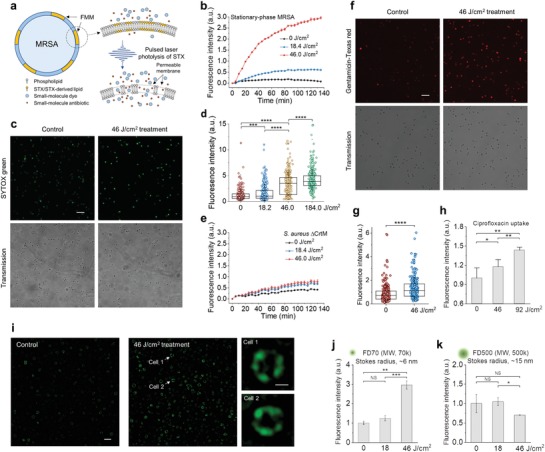
First mechanism for photo‐disassembly of membrane microdomains: membrane permeabilization. a) Schematic of membrane permeability mechanism via pulsed laser photolysis of STX. b) Real‐time intracellular uptake kinetics of SYTOX green by stationary‐phase MRSA (NRS384) with or without pulsed laser treatment. c) Confocal fluorescence images of intracellular uptake of SYTOX green by stationary‐phase MRSA (NRS384) cells with or without pulsed laser treatment. (Top) fluorescence images. (Bottom) corresponding transmission images. d) Statistical analysis of fluorescence signal from MRSA cells in (c) from each treated group with *N* ≥ 300 per group. e) Real‐time intracellular uptake kinetics of SYTOX green by stationary‐phase *S. aureus* ΔCrtM with or without pulsed laser treatment. f) Confocal fluorescence images of intracellular uptake of gentamicin‐Texas red by stationary‐phase MRSA (NRS384) cells with or without pulsed laser treatment. (Top) fluorescence images. (Bottom) Corresponding transmission images. g) Statistical analysis of fluorescence signal of MRSA cells in (f) from each treated group with *N* ≥ 300 per group. h) Fluorescence detection of ciprofloxacin uptake by stationary‐phase MRSA (NRS384) with or without pulsed laser treatment. i) Structured illumination microscopic images of FD500 uptake by stationary‐phase MRSA (NRS384) cells with or without pulsed laser treatment. Insets shows representative images of FD500 distribution on single cell after 46 J cm^−2^ laser treatment. Fluorescence detection of j) FD70 and k) FD500 uptake by stationary‐phase MRSA (NRS384) with or without pulsed laser treatment. MW, molecular weight. Scale bar, 5 µm for (c,f,i) and 0.5 µm for zoom‐in images in (i). *N* = 3 for all the above measurements.

STX is known acting as the constituent lipid of FMM, which are embedded in the lipid bilayer of virulent *S. aureus* strains and implicated in maintenance of membrane integrity. Therefore, we hypothesize that STX photolysis disrupts membrane integrity by increasing membrane permeability, thus facilitating the intracellular accumulation of small‐molecule dyes or antibiotics via passive diffusion (**Figure** **2**a). To prove this point, membrane permeability with or without laser treatment was evaluated in real time by SYTOX green (600 Da), a fluorescent dye for nucleic acids stain of cells only with compromised membrane. With increased laser treatment dose, a significantly larger and faster uptake of SYTOX green by MRSA cells (NRS384) is observed, indicating severely compromised cell membranes; whereas cells without laser treatment show negligible uptake, which validates the role of STX on membrane integrity (Figure 2b). These results were further confirmed by confocal fluorescence imaging and statistical analysis of signal intensity for individual cells. From Figure 2c,d and Figure S2a, Supporting Information, significantly brighter fluorescence signal from the entire cell population is observed over laser treatment dose, indicating different levels of membrane permeability. After 92 J cm^−2^ laser treatment, such damaged membranes are unable to recover even with 2 h culturing (Figure S2b, Supporting Information). In contrast, for *S. aureus* ΔCrtM and log‐phase MRSA (NRS384), no significant difference in SYTOX green uptake is shown between laser treated vs the untreated (Figure 2e and Figure S2c, Supporting Information).

Based on these findings, we further hypothesize that increased membrane permeability induced by STX photolysis would allow passive diffusion of small‐molecule antibiotics that target intracellular activities. To demonstrate this point, we used the aminoglycoside, gentamicin, as an example. Gentamicin was first conjugated with a fluorescent dye, Texas red, and then imaged via confocal fluorescence microscopy after co‐culturing with cells. As expected, MRSA (NRS384) cells with laser treatment accumulate significantly more gentamicin molecules than untreated, from either single cells (Figure 2f,g) or the entire cell population (Figure S2d, Supporting Information). The uptake of ciprofloxacin, another small‐molecule antibiotic that belongs to fluoroquinolone class, can be directly detected via its endogenous fluorescent nature. Compared to the untreated cells, increased fluorescence signal is shown on cells with laser treatment (Figure 2h). These results further confirm that small‐molecule antibiotics can diffuse into the cell via permeable membrane induced by laser treatment.

To estimate how large a molecule can diffuse into the damaged membrane, we applied dextran‐labeled fluorescein isothiocyanate (FITC‐dextran) with variable molecular weight/Stokes radius and monitored its insertion before and after laser treatment. For FD70 with molecular weight of 70k Da and Stokes radius of 6 nm, larger laser treatment dose yields increased fluorescence signal either at individual cell level (Figure 2i) or from the entire cell population (Figure 2j). Laser treatment over 46 J cm^−2^ leads to ample insertion of FD 70 (Figure 2j). Super‐resolution imaging of individual cells further shows that these dyes are primarily inserted and concentrated within FMM (Figure 2i, zoom‐in images). In contrast, when FD500 with molecular weight of 500k Da and Stokes radius of 15 nm was applied, no uptake is shown, indicating an upper limit on pore size of 30 nm level (Figure 2k). These results suggest that after effective STX photolysis, FMM becomes porous, allowing molecules with Stokes radius up to nanometer level to diffuse through or insert into the membrane.

### 2.3 Photo‐Disassembly of FMM Mechanism 2: Membrane Fluidification

**Figure 3:**
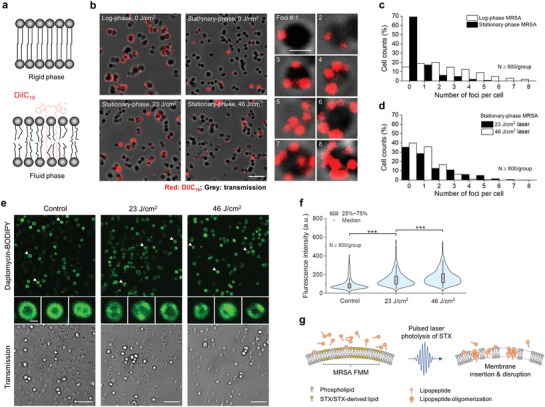
Second mechanism for photo‐disassembly of membrane microdomains: membrane fluidification. a) Schematic of membrane insertion of DiIC_18_ induced by gel/rigid‐to‐liquid/fluid phase change. b) (Left and middle columns) fluorescence images of DiIC_18_ foci formation for groups including log‐phase and stationary‐phase MRSA (NRS384) with or without laser treatment. (Right column) zoom‐in fluorescence images of MRSA cells with different foci number on each cell. Fluorescence from DiIC_18_, red; transmission, grey. Scale bar, 5 µm for (left and middle columns) and 1 µm for (right column). Statistical analysis of foci number on cells from each group in (b): c) log‐phase and stationary‐phase MRSA without laser treatment; d) stationary‐phase MRSA with different laser treatment dose. *N* ≥ 800/group. e) (Top row) fluorescence images of daptomycin‐BODIPY on stationary‐phase MRSA (NRS384) with or without laser treatment. (Middle row) representative zoom‐in images of the upper row. (Bottom row) corresponding transmission channels. Scale bar, 5 µm for (top and bottom row) and 0.5 µm for (middle row). f) Statistical analysis of fluorescence signal intensity from MRSA cells in (e) with or without laser treatment with *N* ≥ 800. g) Schematic of antibiotic membrane insertion mechanism via pulsed laser photolysis of STX.

After effective STX photolysis, its products no longer maintain the chemical structure and properties of STX. The unsaturated tail of STX is truncated as unveiled by Raman spectroscopy results; the polarity of its products becomes significantly higher than that of STX as suggested by liquid chromatography results.^12^ As a result, these products spontaneously tend to disperse or detach from their original membrane organization. These behaviors profoundly disrupt the lipid packing within the microdomain, thus changing the membrane fluidity and subsequently facilitating the insertion of membrane targeting antibiotics, for example, daptomycin. To test this hypothesis, we evaluated the membrane fluidity with or without laser treatment by DiIC_18_, a fluorescent lipophilic cationic indocarbocyanine dye that displays affinity for membrane areas with increased fluidity due to its short hydrocarbon tail^20^ (**Figure** **3**a). As shown in Figure 3b–d, significantly more DiIC_18_ is shown up as foci in log‐phase MRSA (NRS384) when compared to the stationary‐phase, as membrane of stationary phase becomes more rigid than that of log phase, partially due to the presence of rigid STX.^21^ After laser treatment, the foci number on each cell is significantly increased when compared with that of stationary‐phase cells without laser treatment. Notably, 70% of cells in stationary phase show no detectable fluorescence signal, whereas this portion drops dramatically to 35% after 23 J cm^−2^ laser treatment. The ample uptake indicates that laser treatment renders membrane more fluid due to the depletion of rigid unsaturated STX tail and the subsequent loose packing of lipid bilayer.

The increased membrane rigidity by STX overexpression promotes the bacterial resistance against daptomycin, a cationic antimicrobial peptide, by reducing its membrane binding and subsequent membrane disruption.^21^, ^22^ Therefore, we further hypothesize that increased membrane fluidity after STX photolysis facilitates the insertion of daptomycin. To prove this point, we first labeled daptomycin with BODIPY (molecular structure shown in Figure S3a, Supporting Information), then imaged the cellular uptake of daptomycin by MRSA (NRS384) with or without laser treatment. From Figure 3e,f, significantly more daptomycin uptake is shown for laser‐treated groups when compared to the untreated groups; longer treatment yields higher uptake. More interestingly, daptomycin distribution between laser‐treated and untreated groups is quite different; for the untreated, daptomycin distributes evenly on the cell membrane, whereas, aggregates or domain‐like structures with bright signal are found on cells after laser treatment (representative zoom‐in images in the middle row in Figure 3e). These aggregates most likely form within FMM due to the promoted insertion and oligomerization of daptomycin. Collectively, these results provide evidences to support the ample increase of membrane fluidity after STX photolysis, thus potentiating antibiotic lipopeptides to insert and oligomerize within the domains and further disrupt cell membrane as illustrated in Figure 3g.

### 2.4 Photo‐Disassembly of FMM Mechanism 3: Membrane Protein Detachment

**Figure 4:**
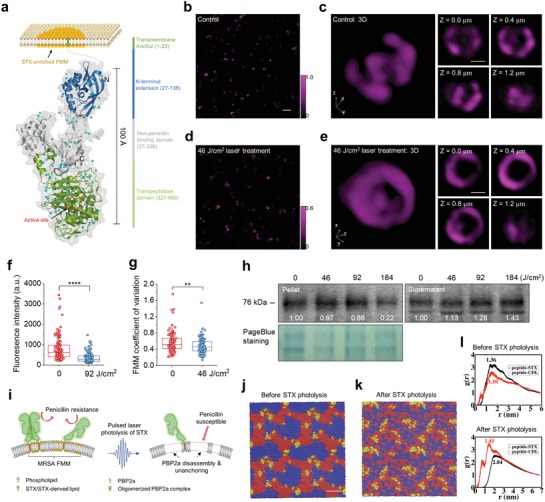
Third mechanism for photo‐disassembly of membrane microdomains: membrane protein detachment. a) Schematic of PBP2a protein structure and location relative to STX enriched membrane microdomain. SIM images of PBP2a via immunostaining on MRSA (NRS384) cells in b) 2D and c) 3D. Intensity color bar applies to (b,c). SIM images of PBP2a immunostaining on MRSA (NRS384) cells in d) 2D and e) 3D after 46 J cm^−2^ laser treatment. Intensity color bar applies to (d,e). Scale bar, 2.0 µm for (b,d) and 0.5 µm for (c,e). f) Statistical analysis of signal intensity from MRSA (NRS384) cells with or without laser treatment with *N* ≥ 100. g) Statistical analysis of PBP2 coefficient of variation on MRSA (NRS384) cells with or without laser treatment with *N* ≥ 100. h) Western blot of PBP2a on MRSA (NRS384) pellets and its supernatant for groups with different laser treatment dose. Numbers indicate the integrated signal intensity. Pageblue staining of the same samples was used as a loading control. i) Schematic of PBP2a disassembly and detachment mechanism via pulsed laser photolysis of STX. j) Self‐assembled microphase separated domain structures of modeled membrane after 10 µs molecular dynamics simulation. Full‐length STX lipids, red; cardiolipin lipids, blue; PBP2a peptides, yellow. k) Final configuration of modeled membrane with truncated STX after 10 µs molecular dynamics simulation. Color scheme also applies to (j). Water and ions are made invisible for clarity for (j,k). Scale bar, 5 nm for (j,k). l) RDFs of PBP2a peptides relative to the full‐length STX and cardiolipins (upper panel) and truncated STX and cardiolipins (lower panel). Numbers on the plot indicate the locations of the first peak for each RDF.

To demonstrate how STX photolysis further malfunctions membrane proteins that are co‐localized within STX‐enriched FMM, we chose penicillin‐binding protein 2a, PBP2a, as an example. MRSA acquires resistance to beta‐lactam antibiotics through expression of PBP2a, a protein[qv: 2a] that primarily anchors within FMM through its transmembrane helix and hides its targeting site inaccessible by beta‐lactam antibiotics (**Figure** **4**a). Considering the relative structural organization of STX and PBP2a, we hypothesize that PBP2a protein complex can be disassembled and unanchored from cell membrane upon effective STX photolysis. To validate this point, we first resolved the structural distribution of PBP2a under a structured illumination microscopy via immunostaining MRSA (NRS384) cells with anti‐PBP2a antibodies both for laser‐treated (Figure 4b,c) and the untreated (Figure 4d,e). For the untreated, we observed bright fluorescence signal from all stationary‐phase MRSA cells due to ample PBP2a expression. These proteins are accumulated discretely within small membrane domains as visualized in both 3D (Figure 4b) and 2D along various depths (Figure 4c). Three to four foci on average is found on each cell, indicating the prevalence of microdomain formation when cells reach their stationary phase (Figure S4a, Supporting Information). Once treated with pulsed laser, dramatically decreased signal intensity and altered signal distribution are observed on each individual cell (Figure 4d,e). Laser‐treated cells have around two times lower signal intensity when compared with the untreated, thus indicating a large portion of PBP2a proteins are detached from cell membrane (Figure 4f). The left PBP2a proteins are dispersed laterally with its dispersion quantified by coefficient of variation, which is significantly higher than that of the untreated (Figure 4g, quantification method shown in Figure S4b, Supporting Information). Such detachment and dispersion lead to significantly reduced contrast between FMM and its neighboring lipid bilayer (Figure 4e). Western blotting results further confirms the PBP2a detachment mechanism, as increased amount of PBP2a is found in supernatant over laser treatment dose, whereas decreased amount found in MRSA pellets (Figure 4h). Taken together, photolysis of the constituent lipids leads to disassembly and detachment of PBP2a from FMM, thus disables MRSA's defense to penicillins as illustrated in Figure 4i. Additionally, as PBP2a is primarily utilized to catalyze cell‐wall crosslinking, their detachment further affects cell wall synthesis and potentially cell viability.

To further investigate the membrane phase and its mechanical properties, we built a coarse‐grained membrane model that contains STX, cardiolipin lipids, and transmembrane helixes of PBP2a proteins (coarse‐grained representations shown in Figure S4c–f, Supporting Information) and performed microsecond‐scale molecular dynamics simulations. At the initial simulation configuration, STX, cardiolipin lipids, and peptides randomly disperse in the built bilayer (Figure S4g, Supporting Information). During 10 µs simulation, these molecules spontaneously self‐assemble to a microphase separated system containing well distinguishable STX and cardiolipin microdomains, despite cardiolipin being a charged lipid; PBP2a peptides localize to the center of STX domains or the vicinity of STX/cardiolipin domain interface (Figure 4j). The formation of microdomain is primarily driven by the preferable interactions among lipid tails of similar saturation or unsaturation nature, as in current system all four tails of cardiolipin are saturated, whereas STX lipid has a long unsaturated tail. This result is consistent with lipid domain formation commonly found for systems with a mixture of saturated and unsaturated lipids such as DOPC/DPPC, DOPC/DPPG, DOPG/DPPC, and many others.^23^ To quantify the relative position and abundance of PBP2a peptides relative to STX and cardiolipin lipids, the radial distribution functions (RDFs), *g*(*r*), of PBP2a peptides were calculated. Figure 4l (upper panel) shows that the RDF peak of PBP2a peptide to STX is higher and located at smaller distance when compared to that of PBP2a peptide to cardiolipin, indicating that PBP2a peptides preferentially interact with STX lipids over cardiolipin, due likely to the better packing between the rigid fully unsaturated STX tail and the PBP2a transmembrane helix.

Our Raman spectroscopy results suggest that photolysis of STX leads to the loss of its rigid and unsaturated tail, the conjugated C=C chain. Thus, to mimic the scenario after STX photolysis, we repeated our simulations by replacing full‐length STX with truncated STX with its unsaturated tail removed from the model (Figure S4d, Supporting Information). Interestingly, the truncated STX lipids no longer form microdomains. As a result, all the lipids and PBP2a peptides are randomly dispersed (Figure 4k). Moreover, the RDF of PBP2a peptide to cardiolipin now features a higher peak at a smaller distance than that of PBP2a peptide to STX, suggesting that the PBP2a proteins prefer to interact with cardiolipins over truncated STX (Figure 4l, lower panel). The different phase features before and after STX photolysis also lead to different membrane mechanics. For example, the calculated area expansion modulus (*K_A_*) of the membrane after microdomain formation is ≈58 *k_B_T* nm^−2^, which is significantly higher than the value of ≈42 *k_B_T* nm^−2^ with truncated STX, cardiolipins, and peptides randomly dispersed after STX photolysis. This suggests that following the truncation of the unsaturated STX tail, the membrane loses the microphase separated domain structure and becomes more loosely packed, which in turn likely reduces the affinity of PBP2a protein to the membrane. Collectively, our simulations provide a plausible rationale for the STX photolysis‐induced membrane remodeling, including the loss of functional domains, the increase of membrane permeability and fluidity, and the detachment of PBP2a from the membrane.

### 2.5 Restoration of Conventional Antibiotics

**Figure 5:**
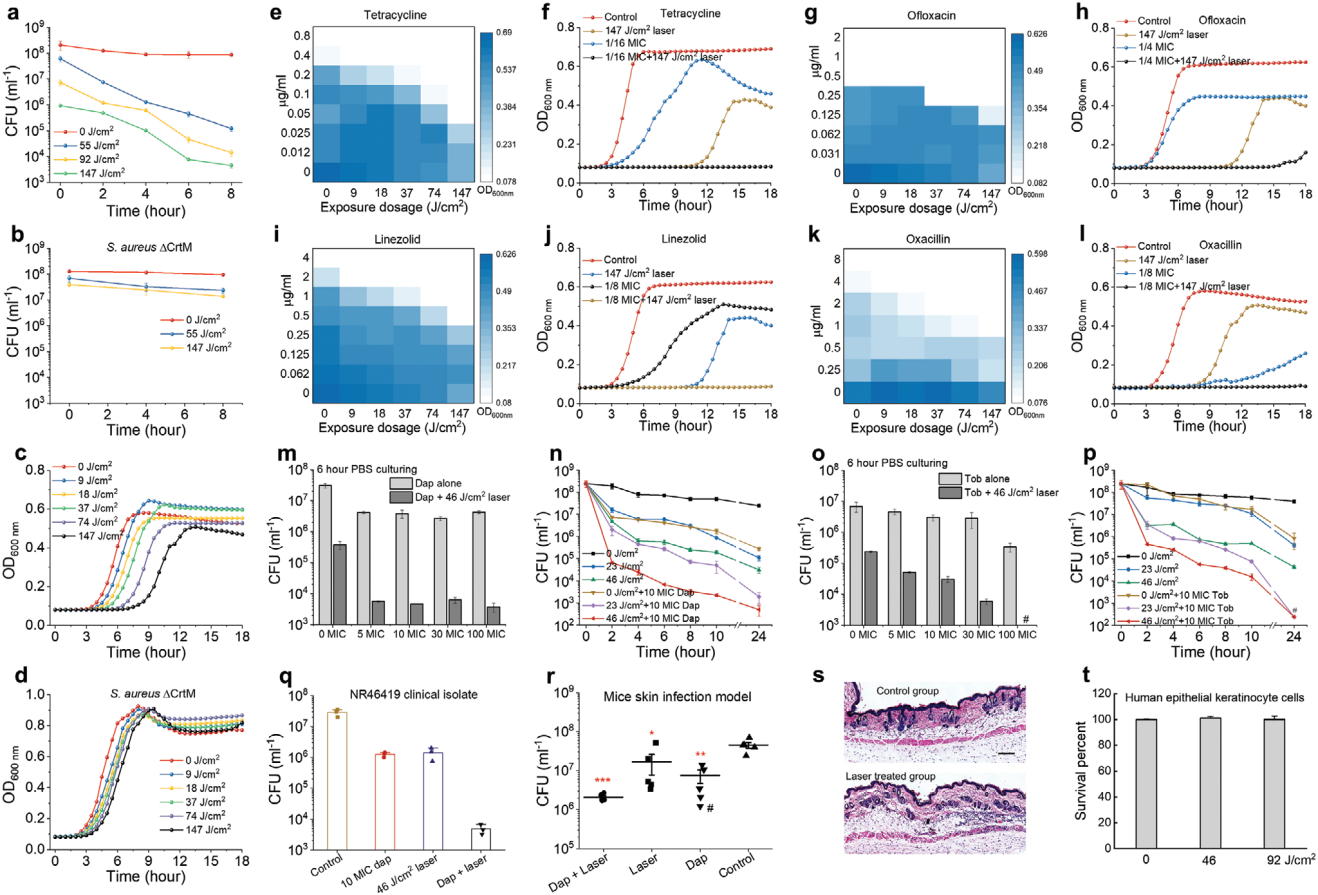
Photo‐disassembly of membrane microdomains restores a broad spectrum of conventional antibiotics. Time‐dependent killing of stationary‐phase a) MRSA (NRS384) and b) *S. aureus* ΔCrtM cells in phosphate‐buffered saline after different laser treatment dose. Post‐exposure effect of stationary‐phase c) MRSA (NRS384) and d) *S. aureus* ΔCrtM after different laser treatment dose. Checkerboard assay results for synergy evaluation on MRSA (NRS384) between laser treatment and different classes of antibiotics: e,f) tetracycline, g,h) ofloxacin, i,j) linezolid, and k,l) oxacillin. f,h,j,l) Selected cell growth curves acquired from corresponding checkerboard assay results of each antibiotic. m) Viability of stationary‐phase MRSA (NRS384) after laser treatment alone or in combination with daptomycin with different concentrations followed by 6‐h incubation in phosphate‐buffered saline. n) Time‐dependent killing of stationary‐phase MRSA (NRS384) in phosphate‐buffered saline after laser treatment alone or in combination with 10 × MIC daptomycin. o) Viability of stationary‐phase MRSA (NRS384) after laser treatment alone or in combination with gentamicin with different concentrations followed by 6‐h incubation in phosphate‐buffered saline. p) Time‐dependent killing of stationary‐phase MRSA (NRS384) in phosphate‐buffered saline after laser treatment alone or in combination with 10 × MIC gentamicin. q) Time‐dependent killing of stationary‐phase VRSA (NR46419) strain in phosphate‐buffered saline for four different treatment groups. r) Efficiency of laser treatment alone or in combination with daptomycin on MRSA (NRS384)‐caused mice skin infection model. # denotes an outlier. s) Hematoxylin and eosin stained histology evaluation of phototoxicity on mice skin. The mice used and treatment procedure applied were the same as that of (r) but without MRSA infection on the skin. t) Viability of human keratinocyte cells over different laser treatment dose to evaluate phototoxicity. *N* = 5 for CFU enumeration for in vivo mice study. Dap, daptomycin; Tob, tobramycin. *N* = 3 for the rest CFU enumeration, for checkerboard assay of each antibiotic and for phototoxicity evaluation on both human cells and in vivo mice.

With cell membrane catastrophically damaged via STX photolysis, we further reasoned that both cell growth and cell viability are severely compromised by laser treatment alone. To test this point, time‐killing assay in phosphate‐buffered saline was first performed on stationary‐phase MRSA (NRS384) cells with or without laser treatment. Compared with the untreated, laser‐treated cells are killed quickly and efficiently due to their disassembled FMM and incapacity for recovery (**Figure** **5**a). The killing efficiency shows strong illumination dose/time dependence; 147 J cm^−2^ (16 min) laser treatment yields nearly 5‐log killing when compared to the untreated. In contrast, *S. aureus* ΔCrtM shows relatively negligible killing by laser treatment (Figure 5b). These results confirm that STX photolysis induced membrane disruption is the underlying eradication mechanism. Additionally, its recovery ability after laser treatment was assessed via a post‐exposure effect assay, similar to post‐antibiotic effect,^24^ as an important way to establish the optimal dosing regimen. The post‐exposure effect of stationary‐phase MRSA (NRS384), depending on STX expression condition and laser treatment dose, reaches up to 6–9 h, due primarily to the membrane disruption mechanisms (Figure 5c and Figure S5a, Supporting Information), whereas no significant post‐exposure effects were observed for log‐phase MRSA (NRS384) (Figure S5b, Supporting Information) or *S. aureus* ΔCrtM (Figure 5d). This post‐exposure effect indicates a slow recovery for stationary‐phase cells after laser treatment thus fewer doses required for patients, which is superior to the post‐antibiotic effect of most antibiotics including oxacillin, ofloxacin, and gentamicin (<1 h post‐antibiotic effect for all three antibiotics, Figure S5c, Supporting Information). More significantly, STX photolysis‐induced FMM disassembly can pave a new approach to sensitize these bacteria to conventional antibiotics, even by antibiotics presumed to have no activity against MRSA, such as penicillins.

To explore laser‐mediated synergism with antibiotics, we first applied the checkerboard assay as a screening method. Interestingly, synergism is identified between laser treatment and several major classes of antibiotics for MRSA (NRS384) growth inhibition (Figure 5e–l). Using tetracycline as an example, the lowest concentration needed to completely inhibit MRSA growth within 18 h is steadily decreased by increased laser treatment dose; 147 J cm^−2^ (16 min) laser treatment enables a 16‐fold reduction, where two‐fold change or larger is regarded as synergy based on fractional inhibitory concentration index (FICI) (Figure 5e,f). The similar results are found for quinolones: ofloxacin and ciprofloxacin (Figure 5g,h and Figure S5d,e) and oxazolidinone: linezolid (Figure 5i,j) with two‐fold, eight‐fold reduction, respectively. Notably, tetracyclines, oxazolidinones, and quinolones all target intracellular activities; therefore, they have to penetrate through the membrane barrier in order to be functional. These growth inhibition results further validate our hypothesis that photo‐disassembly of FMM renders membrane permeable to allow passive diffusion of small‐molecule antibiotics inside cells, thus increasing their effectiveness against MRSA. Due to the disassembly and detachment of PBP2a proteins on cell membrane, laser treatment further sensitizes MRSA to penicillin: oxacillin with its concentration as low as 1 µg mL^−1^, eight‐fold lower than that of oxacillin‐treated alone (Figure 5k,l). In contrast, when vancomycin, an antibiotic that inhibits cell wall biosynthesis, was tested, no synergism is shown (Figure S5f,g, Supporting Information). For bactericidal antibiotics, time‐killing assay was then applied as the screening method. Due to laser‐mediated membrane insertion and further disruption, 10 × MIC daptomycin is found capable of eradicating stationary‐phase/dormant MRSA (NRS384) cells synergistically with only 46 J cm^−2^ (5 min) laser treatment (e.g., more than 3.5‐log reduction after 6 h), whereas antibiotics alone show very limited killing even at 100 × MIC (e.g., 1‐log reduction after 6 h) (Figure 5m,n). The similar synergistic killing is observed for aminoglycoside: tobramycin (Figure 5o,p) due to its passive diffusion via laser‐mediated permeable membrane. The synergistic therapy between 10 × MIC daptomycin and laser treatment are also effective in eradicating VRSA (NR46419) and multidrug‐resistant MRSA clinical isolates (Figure 5q and Figure S5h,i, Supporting Information). Additionally, the synergy with laser treatment for MRSA killing is not only limited to conventional antibiotics; laser treatment facilitates human whole blood by both killing and inhibiting cell division for stationary‐phase MRSA (NRS384) (Figure S5j, Supporting Information); ROS‐producing agents, for example, hydrogen peroxide (at 220 µm low concentration) synergizes with laser treatment and kills stationary‐phase MRSA (NRS384) by 4 log within 2 h, whereas hydrogen peroxide alone shows minor killing even at 22 mm high concentration (Figure S5k, Supporting Information). In these cases, besides membrane disruption mechanisms, the depleted antioxidant function of STX contributes to ROS‐based killing, consistent with previous findings.^8^

To determine the clinical relevance of the synergistic therapy between laser treatment and conventional antibiotics, the last‐resort antibiotic, daptomycin, was used as the example and further applied on in vivo mice skin infection models. To compare the efficacy of different treatment schemes, four groups (control group, 10 mg mL^−1^ daptomycin‐treated group, 92 J cm^−2^ (10 min) laser‐treated group, and 10 mg mL^−1^ daptomycin plus 92 J cm^−2^ laser‐treated group) were applied following a four‐day treatment protocol as designed in Figure S5l, Supporting Information. After the treatment regimen, infected tissue for each mouse was collected with bacterial load quantified via colony‐forming unit (CFU) enumeration. The CFU statistical results for each treatment group (Figure 5r) suggest that laser alone‐treated group and daptomycin alone‐treated group enable 58% and 81% cell killing, respectively; whereas daptomycin plus laser treatment kills around 95% of MRSA in infected skin area. Additionally, the wound areas treated by laser plus daptomycin appear healthier and show the trend of recovery when compared to other groups, as these wound areas show significantly less purulent material, swelling and redness around the edge of the wound. To further evaluate the potential phototoxicity in in vivo model, we followed the same treatment protocol as mice skin infection model except removing the MRSA injection step. After the treatment, the skin regions of interest were collected and analyzed via hematoxylin and eosin stained histology slides (representative images shown in Figure 5s). As expected, no phototoxicity‐induced structure change is observed in the laser‐treated group. Additionally, the viability of human epithelial keratinocyte cells is also not affected by laser treatment, even under high laser dose (Figure 5t).

### 2.6 Inhibition of Antibiotic Resistance Development

**Figure 6:**
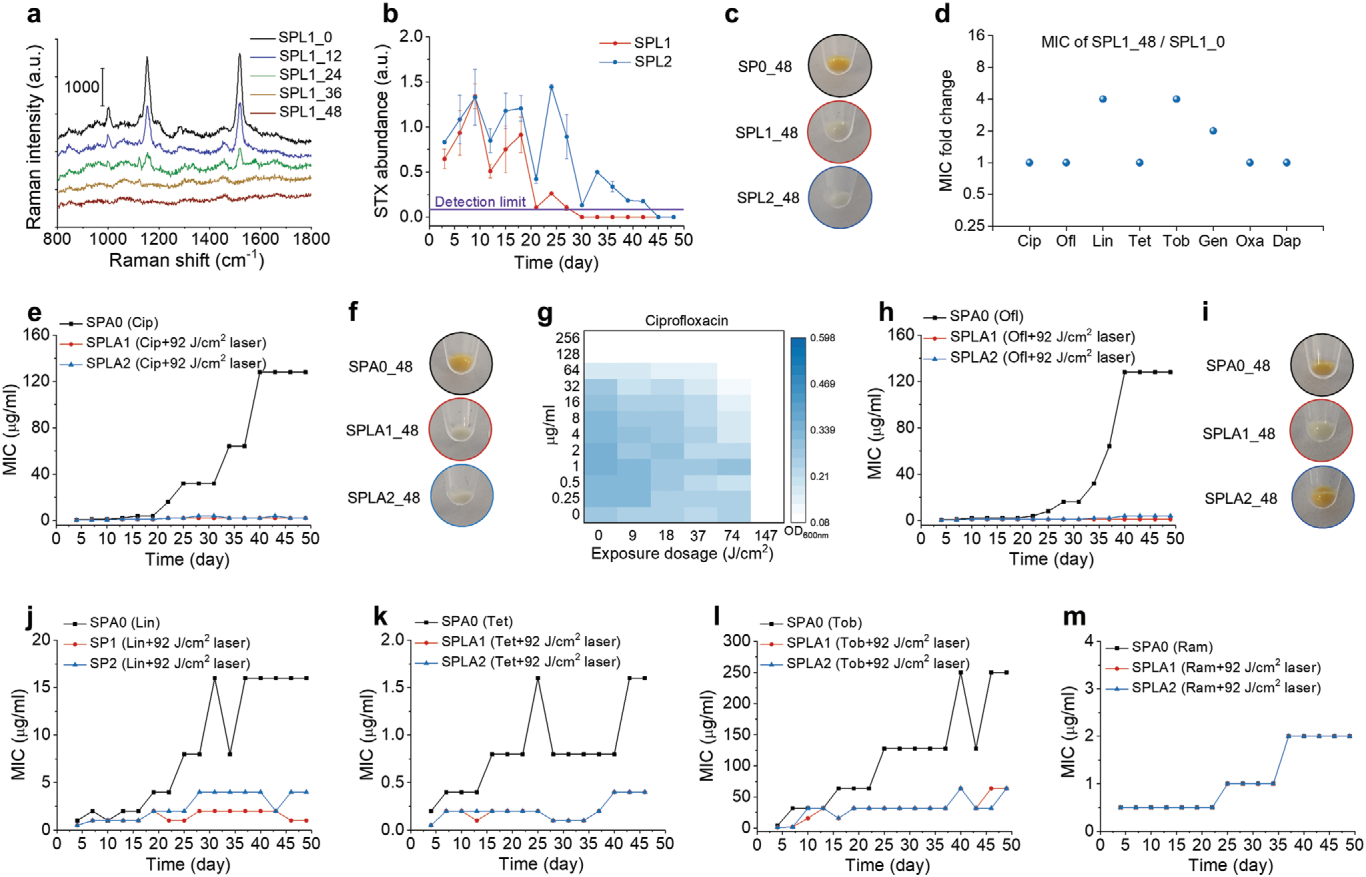
Photo‐disassembly of membrane microdomains inhibits resistance development to conventional antibiotics. a) Representative resonance Raman spectroscopy of STX in stationary‐phase MRSA (NRS384) cells at different time checkpoints for the group treated with 92 J cm^−2^ (10 min) laser alone over 48‐day serial passage. b) STX abundance in stationary‐phase MRSA (NRS384) cells over 48‐day serial passage for groups with or without 92 J cm^−2^ (10 min) laser alone quantified via Raman peak amplitude at 1161 cm^−1^. c) Images of spun‐down cells in (b) after 48‐day serially passage showing STX pigmentation. d) MIC fold change of SPL1 for different classes of antibiotics after 48‐day serial passage. Resistance acquisition for MRSA (NRS384) over 48‐day serial passage in the presence of sub‐MIC levels of antibiotics with or without 92 J cm^−2^ (10 min) laser treatment: e) ciprofloxacin, h) ofloxacin, j) linezolid, k) tetracycline, l) tobramycin, m) ramoplanin. f,i) Images of spun‐down cells from (e) and (h), respectively, after 48‐day serially passage showing STX pigmentation. g) Checkerboard assay of SPA0_48 showing that 147 J cm^−2^ (16 min) laser treatment completely eliminated cell growth. *N* = 3 for checkerboard assay study, Raman spectra, and STX quantification. SPO, serial passage without any treatment; SPL1 and SPL2, serial passage in independent duplicate with laser treatment alone; SPA0, serial passage with sub‐MIC antibiotic treatment alone; SPLA1 and SPLA2, serial passage in independent duplicate with 92 J cm^−2^ laser plus sub‐MIC antibiotic treatment. The numbers after these abbreviations denote serial passage days.

To study MRSA response to our phototherapy, we monitored STX expression level of MRSA (NRS384) during 48‐day serial passage study for 92 J cm^−2^ (10 min) laser alone‐treated group. Over the course of 48‐day passage, steadily decreased STX expression is observed for laser alone‐treated group, as resonance Raman peaks for STX drops over serial passage (**Figure** **6**a,b); on the 30th and 45th day for two independent replicates, STX abundance drops below the detection limit (Figure 6b); the color of the spun‐down cells for both replicates turns to purely white on 48th day whereas the color of the untreated kept golden (Figure 6c). Plate inoculation results further confirm that there is no single colony expressing STX pigment for both replicates after 48‐day passage. These results suggest that STX virulence can be eliminated by serial laser treatment without any resistance development. When compared with the original MRSA, the susceptibility of this new phenotype to different antibiotics shows no change or only minor change after serial treatment (Figure 6d).

We then studied the development of resistance for different antibiotics with or without 92 J cm^−2^ (10 min) laser treatment in parallel by monitoring MICs for each group in the presence of corresponding antibiotic at sub‐MIC level over the course of 48‐day passage. Strikingly, in the presence of laser treatment, ciprofloxacin‐treated group shows no resistance over the entire passage study, as its MIC is kept ≤2 µg mL^−1^, whereas the MIC of ciprofloxacin alone‐treated group has reached 128 µg mL^−1^, 256‐fold increase relative to its starting MIC (Figure 6e). Its spun‐down cells turn purely white for both replicates (Figure 6f); plate inoculation results show that one replicate has no STX expression and the other with mixture of golden and white colonies, consistent with STX expression level monitored through resonance Raman spectroscopy (Figure S6a, Supporting Information). These results suggest that STX virulence is closely related to ciprofloxacin resistance development via overexpression of efflux pumps;^25^ depletion of STX completely inhibits ciprofloxacin resistance. Therefore, it is highly possible that efflux pump proteins are co‐localized within STX‐enriched FMM and that STX photolysis malfunctions these efflux pumps. Interestingly, using checkerboard assay on the ciprofloxacin‐resistant MRSA (MIC: 128 µg mL^−1^), we found that 147 J cm^−2^ (16 min) laser treatment alone completely inhibits the growth of these cells (Figure 6g). This phenomenon suggests that the survival of ciprofloxacin‐resistant MRSA relies heavily on STX expression to promote efflux pumps. To further explore this class of antibiotics, ofloxacin was investigated in the serial passage study. Similar results are achieved as shown in Figure 6h,i. After a 48‐day serial passage, MIC of ofloxacin alone‐treated group reaches 128 µg mL^−1^, whereas ofloxacin plus laser‐treated replicates have MICs of 1 and 4 µg mL^−1^, respectively. Based on plate inoculation results, one replicate has pure white colonies and the other had a mixture of white and golden colonies. Together, these results suggest STX photolysis not only increases the susceptibility of MRSA to fluoroquinolones, but also inhibits its resistance development.

Laser‐mediated resistance inhibition is also found for other antibiotic classes that synergize with STX photolysis, including linezolid, tetracycline, and tobramycin (Figure 6j–l). Delayed resistance development is shown for oxacillin and gentamicin during early serial passages (Figure S6b,c, Supporting Information). In contrast, decreased resistance development is not shown for ramoplanin, a drug that targets cell wall biosynthesis (Figure 6m), as it is not closely related to the membrane disruption mechanisms. Collectively, these results further unveil the causality between STX virulence and antibiotic resistance, as well as demonstrating a way to inhibit resistance development to several major classes of antibiotics via photo‐disassembly of FMM.

## 3 Discussion

Current antimicrobial development pipeline has failed to meet the growing needs of new and effective antibiotics to fight bacterial infections.^5^ Here, we demonstrate a phototherapy approach to combat MRSA antibiotic resistance by targeting STX, a virulence factor as well as an endogenous chromophore residing in *S. aureus* cell membrane. Although antimicrobial effect of blue light has been documented for decades,^26^ the underlying mechanism is still a mystery and its treatment efficacy is limited, hampering its clinical applications. Our studies reported here and recently^12^ show that STX is the molecular target of photons and subject to photolysis in the entire blue range. This finding challenges the traditionally well accepted hypothesis of blue light‐sensitive endogenous porphyrins, meanwhile, profoundly opens new opportunities in this field. The detailed study of STX photochemistry and its photolysis kinetics further suggest a short‐pulsed laser to nonlinearly accelerate STX photolysis efficiency, speed, and depth, that are beyond the reach of low‐level light sources.

Our data show that STX photolysis disorganizes and malfunctions membrane for antibiotic defense in three distinct aspects. First, the disruption renders the membrane more permeable to antibiotic that target intracellular activities, for example, fluoroquinolones and aminoglycosides. Second, membrane becomes more fluid that facilitates the membrane insertion of membrane targeting antibiotics, for example, daptomycin. Third, proteins, for example, PBP2a, that anchors within the FMM is detached and malfunctioned to defense penicillins. These membrane disruption mechanisms revive a broad spectrum of conventional antibiotics to combat MRSA. Additionally, an alternative mechanism underlying the synergy between STX photolysis and fluoroquinolones and tetracyclines could be that photo‐disruption of FMM malfunctions the efflux proteins. Our serial passage data supports this mechanism, as in those cells, the membrane is intact. Along this direction, photodisassembly of FMM could be further extended to revive chloramphenicol, as its resistance is primarily due to the overexpression of *norA*‐encoded efflux pumps within the microdomains.^27^ Collectively, the above four mechanisms to tackle antibiotic resistance are directly related to the laser‐mediated membrane disruption. Further studies could be pursued to explore the interference of such membrane disruption on other antibiotic resistance strategies developed by *S. aureus*, for example, enzymatic inactivation and target alteration.

STX‐targeted phototherapy has shown promising potential as a novel treatment platform. Future studies can examine synergies with other classes of antibiotics, as well as the host innate immune system, and/or other ROS. As STX has the antioxidant function to shield MRSA from ROS attack, effective STX photolysis could further render MRSA susceptible to oxidative host killing including macrophage cells and neutrophils.^12^ Similar to daptomycin, the modulation on cell membrane fluidity via laser treatment can facilitate non‐oxidative host defense of cationic antimicrobial peptides.^21^ Moreover, this platform can be further exploited to screen lead compounds, particularly for those with intracellular targets. Toward the clinical translation of this technology, more follow‐up studies are indispensable regarding the involvement of host immune system, STX expression heterogeneity in vivo, and treatment depth for human, as these factors all play significant roles on the treatment outcome. It is intriguing that *S. aureus* cells without or with minimal STX expression (including these rapidly dividing cells) are much easier to be eradicated by immune clearance or antibiotics,^8^ while *S. aureus* cells with ample STX expression are more susceptible to our phototherapy approach. Furthermore, oxidative stress induced by the host response contributes to the selection of the pigmented cells; thus, strong synergy between the host defense and laser treatment are expected, yet to be studied. Nevertheless, our in vivo mice skin abscess model has shown that laser treatment alone can reduce MRSA burden significantly, and that much more reduced bacterial load are reached via the synergy between laser treatment and daptomycin. Noteworthily, our photodisassembly approach is fundamentally different from photodynamic therapy,^28^ as it relies on bleaching of endogenous STX to disrupt cell membrane, thus specifically targeting *S. aureus*, instead of using externally administrated photosensitizer‐induced ROS for unselective bacterial eradication.

Targeting MRSA STX virulence by photons exemplifies the approach that utilizes the photochemistry between photons and endogenous chromophores to develop a phototherapy platform for bacterial infections. Although, STX molecules are exclusively found in *S. aureus*, we note that carotenoids that have structural and functional similarity present in other bacterial and fungal species,[qv: 22b] thus can be photochemically decomposed or modulated in a similar manner. One such example is granadaene in Group B *Streptococcus*, where the pigment granadaene has a polyene structure similar to STX.^29^ General speaking, pigmentation is a hallmark for many pathogenic microbes; these pigments similarly promote microbial virulence and exhibits pro‐inflammatory or cytotoxic properties.[qv: 3b] Therefore, these pigments could be the targets of photons via either photochemistry or photothermal approach. Therefore, phototherapy approaches based on these specific photon‐chromophore interactions could be further explored along this direction.

## Conflict of Interest

The authors declare no conflict of interest.

## Author Contributions

J.H., P.T.D., and L.J.L. contributed equally to this work. J.H., P.T.D., and J.X.C. conceived the project. J.H., P.T.D., L.J.L. designed, organized, performed the studies, and analyzed the data. J.H. proposed and demonstrated the short‐pulsed laser. J.H. designed, performed, and analyzed the Raman measurements. J.H. and P.T.D. designed, performed, and analyzed the membrane disruption fluorescence assays and imaging experiments. J.H., P.T.D., and L.J.L. designed, performed, analyzed the in vitro and in vivo experiments. J.H. and P.T.D. designed, performed, and analyzed the selection of resistant mutants. Q.C. and T.M. designed, performed, and analyzed the molecular dynamics simulations. J.J.L. and P.T.D. performed the western blotting assay. G.Y.L. and E.R.U. provided the bacterial mutants and constructive discussions. M.N.S. provided the clinical bacterial isolates and constructive discussions. Y.W.Z. and S.J. helped in the in vitro studies. C.Z. helped in the Raman measurements. J.X.C. supervised the overall project. J.H., P.T.D., and J.X.C. co‐wrote the manuscript. All the authors contributed to discussing and editing the manuscript.

## Supporting information

Supporting Information
